# Successful Percutaneous Nephrolithotomy in Patients with Immune-Mediated Thrombocytopenia Treated with Romiplostim

**DOI:** 10.1089/cren.2016.0048

**Published:** 2016-04-01

**Authors:** Andrew N. McCall, Johann P. Ingimarsson, Griffin T. Morrisson, Christopher C. Hook, Amy E. Krambeck

**Affiliations:** Department of Urology, Mayo Clinic, Rochester, Minnesota.

## Abstract

***Background:*** Percutaneous nephrolithotomy (PCNL) for large stone burden can be problematic in patients with significant risk of bleeding complications, specifically thrombocytopenia. This report demonstrates effective correction of two patients' thrombocytopenia, subsequently leading to removal of large stone burden through PCNL.

***Case Presentation:*** We present two Middle Eastern patients who presented with medical histories significant for thrombocytopenia, secondary to splenomegaly and hepatic vein thrombosis, and large volume nephrolithiasis. Patient 1 is a 65-year-old female with a right 5 cm stone and a platelet count of 34,000. Patient 2 is a 45-year-old female with a 3 cm left staghorn stone and a platelet count of 44,000. After consultation with hematology, both underwent therapy with prednisone and intravenous immunoglobulin without improvement in their platelet count. They then received 3 μg/kg/dose of romiplostim weekly that improved their platelet counts to 133,000 and 195,000 in 2 weeks, respectively. Patient 1's PCNL was completed in a single-stage procedure with stone-free status shown on CT postoperative day 1. Patient 2 underwent PCNL and a secondary ureteroscopy for residual stone fragments on postoperative day 2. Both patients experienced no complication during the procedure, hospital stay, or postoperative course. Both continued romiplostim for 20 days postoperatively with platelet levels returning to their baseline range after 1 month.

***Conclusion:*** In the appropriately selected patient, romiplostim can correct thrombocytopenia enough to safely and effectively perform PCNL in patients with underlying hematologic disorders. Close coordination between urology and hematology is imperative to ensure an effective outcome in this challenging patient population.

## Introduction and Background

Bleeding risk is an inherent risk to percutaneous nephrolithotomy (PCNL) with 7.8% of patients having significant bleeding at the time of the procedure and 5.7% of patients requiring a blood transfusion.^1^ This risk is significantly increased in patients with platelets <80,000 per microliter, and may therefore be considered a contraindication in patients with thrombocytopenia.^2^ We present two immune-mediated thrombocytopenia (ITP) patients in need of a PCNL for symptomatic stone disease. Romiplostim, a thrombopoietin receptor agonist that stimulates megakaryocytes to produce platelets, was used to increase platelet count before the procedure.

## Case Report

### Case 1

Patient 1 is a 65-year-old Middle Eastern female with a history of chronic hepatitis C, liver cirrhosis, hypersplenism, and ITP who presented with flank pain and a right 5 cm stone status-post temporizing ureteral stent placement at an outside institution. Her platelet count was 34,000 at the time of initial consultation. She was referred for a consultation with hematology and underwent therapy with prednisone and intravenous immunoglobulin (IVIG) without improvement in platelet count. She subsequently received 3 μg/kg romiplostim weekly that improved her platelet count to 133,000 by 2 weeks. At that point, right PCNL was performed. Percutaneous renal access was performed using triangulation technique and fluoroscopic guidance. The tract was dilated to 30F and a 30F access sheath was placed. Rigid nephroscopy showed large staghorn calculus and ultrasonic lithotripsy was used to break up the stone and remove fragments. An 18F re-entry Malecot catheter was left as a drain. The CT scan performed on postoperative day 1 showed no residual fragments and the patient was discharged home on postoperative day 2 and nephrostomy tube was removed on day 13. She experienced no complication during the procedure, hospital stay, or postoperative course. She continued romiplostim for 20 days postoperatively and her platelets returned to her baseline range after 1 month. Since the time of her procedure, she has undergone one additional ureteroscopic stone extraction due to continued stone formation. She currently is stone free after 10 months of follow-up.

### Case 2

Patient 2 is a 45-year-old Middle Eastern female with a history of hypersplenism, ITP, Crohn's disease, osteopenia, and iron-deficiency anemia who presented with flank pain for evaluation of a 3 cm left staghorn stone with a platelet count of 44,000. Hematology was consulted and she was initially treated with prednisone and IVIG without resolution of thrombocytopenia. She was subsequently treated with 3 μg/kg romiplostim weekly for 2 weeks that increased her platelets to 195,000, at which time a left PCNL was performed. Percutaneous access and nephrolithotomy was performed in a similar manner as described for Patient 1. A residual stone fragment was identified on a stone protocol CT scan on postoperative day 1. Therefore, a secondary ureteroscopy was performed on postoperative day 2, retrieving the fragments. A left ureteral stent was left in place for 2 weeks. Removal was uncomplicated. The patient experienced no complication during the procedures, hospital stay, or postoperative course. Her romiplostim was stopped postoperative day 21 and her platelets returned to baseline around 40,000 1 month postoperatively. She continues to be stone free after 5 months of follow-up.

## Discussion

Large renal calculi can pose a significant challenge to the practicing urologist. Whether removal is through PCNL or through staged retrograde ureteroscopic stone extraction, these procedures can be challenging and place patients at high risk. In addition to procedure difficulty, these calculi tend to occur quite frequently in individuals with multiple medical comorbidities, making the overall treatment and management challenging.

The Society of Interventional Radiology guidelines for periprocedural management of coagulation categorizes percutaneous renal access as a procedure with “significant bleeding risk” with platelet count needing to be >50,000.^3^ Most series report combined major and minor complication rates of PCNL around 10% with a mortality rate being 0.05% to 0.3%, with major complications defined as injury to adjacent structures, severe, bleeding, and severe infection/sepsis.^4^ Minor bleeding after nephrostomy tube placement occurs in as much as 95% of cases, and severe postprocedure bleeding necessitating a transfusion was reported to be around 1% to 4%. In certain cases of significant bleeding, coil embolization or even nephrectomy is necessary to control blood loss.^4^ Platelet count is generally measured as a part of the complete blood count with normal adult platelet count being between 150,000 and 450,000 per microliter. Blood count of 10,000 to 30,000 per microliter of blood can lead to bleeding with minimal trauma and a platelet count of <5000 per microliter poses a life-threatening condition of spontaneous bleeding. Patients with underlying thrombocytopenia, defined as platelets <150,000, and large burden nephrolithiasis propose a challenging and complex scenario that requires a collaboration between multiple specialties to treat.^5^

ITP occurs because of accelerated platelet destruction that is mediated by antibodies to platelets, and decreased platelet production by antibodies directed against megakaryocyte antigen coupled with T-cell suppression. Treatment has historically had low success rates, with one-third of patients failing first-line and subsequent therapies for correction.^6^ First-line therapy includes corticosteroids, anti-immunoglobulin, and IVIG and second-line therapies include azathioprine, rituximab, or splenectomy. First-line treatment generally takes weeks and second-line therapies with long-term success are observed in 15% to 20% of patients at 5 years.^7^ These therapies also come with significant risks to the patient that span from hemolytic anemia to increase risk of infection.^7^ A new second-line agent, romiplostim, is a thrombopoietin receptor agonist that stimulates megakaryocytes to produce platelets at a more rapid rate than normal, in turn overwhelming the immune system's ability to destroy them.^8^ It can produce a platelet response in ∼80% to 90% of patients with ITP.^9^ The typical starting dose is 1 μg/kg, but these doses can be titrated pending subsequent results with increasing platelet counts starting as soon as 2 week after initiation of therapy.^10^ It is known that long-term treatment may be required to maintain these platelet responses, but long-term data are not available. It is known that the positive response on platelet levels can be maintained as long as medical therapy with romiplostim is continued over a short time period.^9,11^ Most common side effects of romiplostim are headache and fatigue with total series adverse events having an incidence of 1% with the most common being thrombocytopenia.^11^ Our case report demonstrates effective correction of two patients' underlying ITP with use of romiplostim, which allowed PCNL to be performed without major postoperative bleeding event. Neither of our patients experienced adverse side effects from the medication.

## Conclusion

In the appropriately selected patient, romiplostim can correct thrombocytopenia to safely and effectively perform PCNL in patients with underlying hematologic disorders. Close coordination between urology and hematology is imperative in ensuring an effective outcome in this challenging patient population.

## Abbreviations Used

CT: computed tomography
ITP: immune-mediated thrombocytopenia
IVIG: intravenous immunoglobulin
PCNL: percutaneous nephrolithotomy
